# Induction role of chitosan nanoparticles to *Anethum graveolens* extract against food-borne bacteria, oxidant, and diabetic activities *in vitro*

**DOI:** 10.3389/fmicb.2023.1209524

**Published:** 2023-07-04

**Authors:** Abdullah Mashraqi

**Affiliations:** Department of Biology, College of Science, Jazan University, Jazan, Saudi Arabia

**Keywords:** chitosan nanoparticles, dill, extract, antimicrobial, food-borne bacteria, antioxidant, anti-diabetic

## Abstract

Foodborne diseases as well as Foodborne pathogens are a global issue with significant effects on human health and economy. Therefore, several investigators have tried to find new alternative approaches to prevent and control this problem. In this context, the present study aimed to find some possible and effective approaches for controlling food-borne bacteria via Dill (*Anethum graveolens* L.) extract (DE) loaded with chitosan nanoparticles (ChNPs) besides its anti-oxidant and anti-diabetic activities. Flavonoid and phenolic contents of DE were detected by HPLC, indicating the presence of 18 constituents, high content (22526.51 μg/mL) of chlorogenic acid, followed by 2236.21 μg/mL of coumaric acid and 2113.81 μg/mL of pyrocatechol. In contrast, low contents of cinnamic acid, methyl gallate, apigenin, daidzein, quercetin, syringic acid, and kaempferol were detected. *B. cereus*, *Staphylococcus aureus, E. coli, S. typhi, E. faecalis*, and *C. albicans* were highly inhibited by DE loaded ChNPs (DELChNPs) with inhibition zones (IZs) of 28.50 ± 0.87, 30.33 ± 0.58, 29.33 ± 0.58, 23.17 ± 0.76, 25.76 ± 0.58, and 24.17 ± 0.29 mm with MIC 15.41 ± 0.36, 7.70 ± 0.17, 15.58 ± 0.07, 31.08 ± 0.07, 31.04 ± 0.07 and 62.33 ± 0.29 μg/mL compared with inhibitory activity caused by DE, where the IZs were 25.83 ± 1.44, 29.67 ± 0.85, 24.83 ± 0.76, 20.33 ± 1.53, 21.17 ± 0.29, and 19.67 ± 1.15 mm with MIC 62.33 ± 0.29, 31.08 ± 0.07, 62.50 ± 0.29, 31.08 ± 0.07, 31.04 ± 0.07, and 249.0 ± 1.73 μg/mL, respectively. Also, the minimum bactericidal concentration (MBC) of DELChNPs was less than DE against all tested microorganisms. The MBC/MIC index documented that DELChNPs were more effective than DE. The biofilm of tested bacteria was inhibited by DE and DELChNPs but with different levels of anti-biofilm activity. For example, the anti-biofilm activity was 79.26 and 86.15% against *B. cereus* using DE and DELChNPs, respectively. DELChNPs and DE, compared with the ascorbic acid, exhibited DPPH scavenging % with IC^50^ values of 7.8 μg/mL, 13.96 μg/mL, and 4.83 μg/mL, respectively. Anti-diabetic activity *in vitro* via inhibition of amylase indicated that IC^50^ was 164.2 μg/mL and 164.3 μg/mL using DE and DELChNPs, respectively. This investigation highlights the vital DE phytoconstituents, particularly DELChNPs which possess important therapeutic effects against food-borne microorganisms and could be utilized as a safe alternative to synthetic drugs.

## 1. Introduction

Dill (*Anethum graveolens* L.) is an annual herb belonging to the Apiaceae family. Its fresh aerial parts or seeds have been extensively utilized to enhance the flavor of the food as a spice. Numerous investigations have focused on the chemical constituents of dill and biological activities. From the earlier literature, dill is utilized for many purposes, including medicinal or nutritional such as minimization of bad breath, management of digestive disorders, lactation stimulation, lipid-lowering, anti-gastric irritation, antidiabetic, antimicrobial, antioxidant, anticancer, and also known as an anti-inflammatory agent (Jana and Shekhawat, 2010; Ozliman et al., 2021; Peerakam et al., 2022). Selen Isbilir and Sagiroglu (2011) illustrated that the dill plays an important role against oxidative stress and improves biopharmaceutical and cosmetic applicability. In traditional treatment, dill is applied as a diuretic agent and used to solve problems associated with the gastrointestinal tract, such as colic to tract intestinal gas, indigestion, and stomach pain. Oshaghi et al. (2017) documented antiglycation, antioxidant, and hepatoprotective activities of dill leaf extract; therefore, they suggested that dill may be applied as a safe source for diabetes problems and liver poisonousness treatment (Oshaghi et al., 2017). Other biological activities were associated to dill extract to manage several diseases such as spasmodic, diabetes, hypercholesterolemia, cancer, and ulcer (Chahal et al., 2017). In the food industry, addition of dill to Kareish cheese documented its efficacy against coliforms, yeast, molds, and *S. aureus*, besides the addition of dill is acceptable to the consumer (Wahba et al., 2010). Recently, Peerakam et al. (2022) tested the crude dill extracts against tyrosinase and collagenase activities. These enzymes responsible for pigmentation and wrinkles revealed an inhibitory influence on collagenase and anti-protein denaturation was observed as a result of dill extracts application. Strong inhibitory action was recorded against *Pseudomonas aeruginosa*, *Escherichia coli*, and *Staphylococcus aureus*. In contrast, moderate inhibitory activity was recorded against *Alternaria alternata* and *Fusarium graminum* using the essential oil of *A. graveolens* (Benlembarek et al., 2022).

Essential oil of *A. graveolens* exhibited growth inhibition of food borne-bacteria including *Salmonella typhi, Enterococcus faecalis, Klebsiella pneumoniae, S. epidermidis*, and *S. aureus*, while *Pseudomonas aeruginosa* and *Escherichia coli* were not inhibited (Tanruean et al., 2014). Essential oil of dill inhibited the growth and conidia germination of *Colletotrichum nymphaeae* by reducing the severity and incidence of anthracnose disease on strawberry fruits (Karimi et al., 2016). Roots extracts, leaves with stems extract, and seeds essential oil of *A. graveolens* were tested against *Aspergillus niger*, *A. flavus*, *A. parasiticus*, *A. ochraceus*, *Penicillium verrucosum*, and *Fusarium graminearum*. The growth of these fungi was inhibited with the reduction of aflatoxin B1 and aflatoxin G1 but mainly using seeds essential oil (Badr et al., 2017). According to several scientific papers, the biological activities of dill were associated with active compounds. α-phellandrene and β-phellandrene were recorded in the essential oil of dill as a main constituent offering antioxidant and antibacterial actions (Peerakam et al., 2014). Catechin, chlorogenic acid, rutin, and quercetin were detected in *A. graveolens* (Ahmed et al., 2021).

In the current decade, nanotechnology, because of its unique characteristics, has attracted the attention of many scientists in various fields, from life to inanimate objects, through the medical, agricultural, food, and electronics fields (Mohamed, 2013; Abdelghany et al., 2018, 2020, 2023; Ganash et al., 2018; Al-Rajhi et al., 2022; Qanash et al., 2023a,b). Even the development of current drugs or increasing their effectiveness has not been devoid of the use of nanomaterials. Also, many plant extracts or their components are loaded or doped with nanoparticles, especially polymer materials such as chitosan (Salehi et al., 2020; Hasanin et al., 2022; Yahya et al., 2022). Compared to bulk chitosan, the unique features of chitosan NPs, such as surface area, the occurrence of active efficient groups, restricted toxicity, and abundant permeability into membranes of cells, have prioritized its possible beneficial applications (Qanash et al., 2023a). Chitosan Nanoparticles serve as an excellent strategy for enhancing the biological activities of several natural compounds as well as the used antibiotics via encapsulating the anti-biofilm agents (Negi and Kesari, 2022). Several scientific reports aimed to improve the efficacy of natural origin constituents in the medicinal and food fields via combination with the chitosan nanoparticles such as marjoram, oregano, savory (Keawchaoon and Yoksan, 2011), *Leucas aspera* (Devi et al., 2015), sage (da Silva et al., 2015), *aloe vera* gel (Yahya et al., 2022), and *Pterocarpus marsupium* (Antunes et al., 2021), all findings of these scientific papers reflected an increase in the activity of these extracts. Depending on the Nanotechnological applications, dill essential oils were encapsulated using copper nanoparticles and tested against plant pathogen *Colletotrichum nymphaeae*, reflecting good promising effectiveness for inhibiting the fungus growth up to 90% at 9 days of treatment with mycelial deformation and twisting of hyphae (Weisany et al., 2019). In another investigation, *Byrsonima crassifolia* essential oils loaded with chitosan nanoparticles reflected good antifungal activities against *Colletotrichum gloeosporioides* and *Alternaria* species compared to bulk oil (Barrera-Necha et al., 2018). With this in mind, identifying phenolic and flavonoid content of the *Anethum graveolens* extract via chromatographic technique, loading the extract with chitosan nanoparticles and its pharmacological properties, including antimicrobial activities against food-borne bacteria, antioxidant, and anti-diabetic activities were all the objectives of the current study.

## 2. Materials and methods

### 2.1. Chitosan nanoparticles

High-quality chitosan nanoparticles (ChNPs) were obtained from Primex, Siglufjordur, Iceland. Analytical grade chemicals, including buffers, solvents, reagents, Dimethyl Sulfoxide (DMSO), and microbial growth media that were utilized in the current research were obtained from the company of Sigma-Aldrich at St. Louis, MS, USA.

### 2.2. Collection of plant material and its extract preparation

**FIGURE 1:**
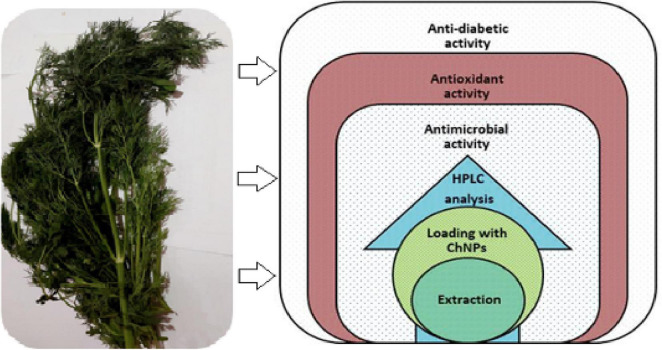
Aerial parts of Dill (*Anethum graveolens*), which underwent successful extraction process and multiple relevant assesses to detect the phytoconstituents, antimicrobial and antioxidant, and anti-diabetic activities.

Shoot systems, including the stem and leaves of *A. graveolens* (Dill) before the flowering period, were collected from a cultivated field in Jazan region, Saudi Arabia. The collected plant was washed with distilled water to remove debris, followed by air-drying at 30°C under shading conditions until a constant weight was obtained. The dried plant sample was ground to fine powder, then extracted using methanol as follows: The powder of the plant (10 g) was extracted with 50 ml of methanol (80%) via an overhead shaker, then centrifuged for 15 min at 10,000 rpm, and the supernatant was re-centrifuged for 10 min at 10,000 rpm to eliminate any suspended particles to obtain clear supernatant. The supernatant was subjected to concentration via evaporation by utilization of a rotary evaporator manufactured at Henan Lanphan Industrial Co., Ltd., China, yielding crude extract 0.5 mg g^–1^, then subjected to further analysis. The extract of Dill plant was subjected to further analysis as summarized in Figure 1.

### 2.3. Identification of phenolic and flavonoid compounds by HPLC

Shimadzu’s High-performance liquid chromatography (HPLC) system, which includes a controller (CBM-20A), two pumps (LC-20AD), a column oven (CTO-20AC), and a photodiode array detector, was used to conduct the HPLC analysis (SPD-M20A). For extract separation, a Vertex Plus column with pre-column (250 × 4 mm, 55 m particle size, packing material ProntoSIL 120-5 C18-H) was employed from Knorr, Berlin, Germany. The extract was diluted (1:2) in 10 L of 80% methanol prior to analysis. The column oven’s thermostat was set to 30°C. Eluents of water (A) and methanol received an addition of 2 mM ammonium acetate (B). The gradient was applied as follows: it began at 10% B, switched linearly to 90% B in 35 min, stayed at 90% for 2 min, switched back to 10% B in 1 min, and then stabilized at 10% B for 2 min. UV/Vis spectra of separated compounds were captured at 190–800 nm. Following HPLC separation, components were added to an AB Sciex Triple TOF mass spectrometer (AB Sciex TripleTOF 4600, Canby, USA) for identification. The negative electrospray ionization temperature was 600°C. The same technique was utilized to prepare the phenolic and flavonoid standards for identification and quantification. Using the PeakView (SCIEX) software, retention time and fragmentation pattern were compared between peaks.

### 2.4. Formulation of ChNPs loaded in DE and TEM characterization of DELChNPs

Chitosan nanoparticles were dissolved in acetic acid at a concentration of 1%, followed by addition of another quantity of ChNPs was added to obtain a final concentration of 2% (w/v). Then glycerol was added at a concentration of 2% (w/v) as a plasticizer agent. To improve the adhesion and wettability characteristics of the prepared solution, tween 20 (0.05%, v/v) was added to the solution. For loading the DE in ChNPs, the DE was added to ChNPs under stirrer condition for 25 min to get 10 % as a final concentration (Torlak and Sert, 2013). Morphological and size of created ChNPs loaded in DE (DELChNPs) were characterized using a transmission electron microscope (TEM) (JEOL-JEM-Plus-1400, Japan). DE and DELChNPs as tested samples were used for further analysis.

### 2.5. Susceptibility test for determining antimicrobial activity and MIC

The susceptibility test was conducted in accordance with NCCLS guidelines. The well diffusion assay was used to conduct a screening test for the inhibition zone. Colonies grown overnight on an agar plate were used to make the inoculum suspension, which was then added to Mueller-Hinton (MH) broth (yeast utilizing malt broth). MH agar plates were inoculated with the suspension using a sterile swab (Yeast utilizing malt agar plates). Dimethyl Sulfoxide (DMSO) was used to dissolve the extract (100 μL of 100 μg/mL). After 24 h at 37°C, the inhibition zone around each well was measured. The DMSO controls were carried out correctly (Qanash et al., 2023a). Five microbial strains, including *Bacillus cereus*, *Staphylococcus aureus* (ATCC 6538), *Escherichia coli* (ATCC 8739), *Enterococcus faecalis* (ATCC 10541), and *Candida albicans* (ATCC 10221), were used as a test organism to determine the inhibitory activity of tested samples. Micro-dilution broth approach was utilized to decide the minimal inhibitory concentration (MIC) (the lowest concentration of the pattern that inhibits the boom of the examined microorganisms). Stock concentrations were used from the tested samples to make a couple of dilutions (0.98–1,000 μg/mL) of the tested samples. A constant quantity (200 μL) of every dilution was used to deliver specified well/s of a sterile 96-well plate comprising broth medium. A 0.5 McFarland suspension of every examined tested bacteria and yeast was mixed with 0.85% sterile normal saline of sodium chloride. Two μL of the microbial suspension [having 2 × 10^8^ bacterial colony forming unit (CFU)/mL, 2 × 10^6^ CFU/mL for yeasts] were transmitted to distinctive well/s. Subsequently, plates were incubated at the same conditions for bacteria and yeast as mentioned above. Finally, MIC values were recorded primarily based on a visible commentary of the microbial growth (Yahya et al., 2022).

### 2.6. Assessment of MBC

A pure subculture of tested microbes was grown overnight, then diluted in growth-supporting broth MH to a dosage of 10^5^–10^6^ CFU/mL. An inventory dilution of the samples is made at hundred instances of the MIC. Further, 1:1 dilutions are made in ninety-six microtiter plates. All dilutions of the DE are inoculated with equal volumes of the targeted microbes. A positive and negative control well is included for every targeted microbe to reveal adequate growth of microbial growth over the progress of the incubation length and media sterility, respectively. An aliquot of the positive control is plated and utilized to set up a baseline level of the used microorganism. Then, the microtiter plates were incubated at the appropriate conditions. The Turbidity shows the growth of the microorganism and the MIC is the lowest concentration. The viability of the minimum bactericidal concentration (MBC) is determined by the dilution representing the MIC and at least two more concentrated test product dilutions. The lowest concentration that shows a pre-determined reduction in CFU/ml is the MBC. The characteristics of tested samples regarding cidal or static were detected through the calculation of the proportions of the MBC/MIC index; if the ratio of MBC/MIC is no great than 4 times the MIC, the DE has cidal effectiveness (French, 2006).

### 2.7. Anti-biofilm activity

The impact of the tested samples on biofilm formation used to be evaluated in 96-well polystyrene flat-bottom plates. Briefly, 300 μL of the inoculated trypticase soy yeast broth (TSY) (10^6^ CFU/mL) were added to every well of microplate, then supplemented with the sub-lethal concentrations (75, 50, and 25%) of MBC in the earlier records. Wells encompassing medium and these except extracts and solely with methanol have been utilized as controls. Plates were incubated at 37°C for 48 h. Once the incubation period ended, the supernatant was eliminated and every well was washed absolutely with sterile distilled water to take away free-floating cells; thereafter, plates were air-dried for 25 min and the biofilm formed was stained throughout 15 min at 30°C with 0.1% aqueous crystal violet. After incubation, the extra stain was eliminated by washing the plate in three instances with sterile distilled H_2_O. As a final point, the bound dye to the microbial cell was solubilized by introducing 250 μL of 95% ethanol to every well for 15 min. Absorbance (Ab) was measured via a reader of microplate at 570 nm wavelength (Antunes et al., 2010). The biofilm inhibition was recorded using the following formula:


$$Biofilminhibition\left(\%\right)=1-\left(\frac{S\,a\,m\,p\,l\,e\,A\,b-B\,l\,a\,n\,k\,A\,b}{C\,o\,n\,t\,r\,o\,l\,A\,b-B\,l\,a\,n\,k\,A\,b}\right)\times 100$$

Blank was signified Ab of growth media only, sample was signified Ab of test microbes after treatment, while Control signified Ab of test microbes lacking any treatment.

### 2.8. Antioxidant activity via free radical scavenging activity

Different concentrations (1.95–1,000 μg/mL) of methanolic extracts of tested samples (2.5 mL) were added to 0.3 mM ethanolic solution (1 mL) of DPPH. The values of absorbance (Ab) were recorded at 517 nm on a Unicam UV/Vis spectrophotometer after 30 min at 25°C in dark. A blank was created using ethanol (1 mL) and plant extract solution (2.5 mL), and a control of DPPH solution and methanol (Qanash et al., 2023a). The following equation was utilized to estimate the % of antioxidant activity:


DPPHradicalscavengingcapacity(%)

$$=1-\left(\frac{S\,a\,m\,p\,l\,e\,A\,b-B\,l\,a\,n\,k\,A\,b}{C\,o\,n\,t\,r\,o\,l\,A\,b}\right)\times 100$$

### 2.9. *In vitro* α-amylase inhibitory activity

The 3,5-dinitrosalicylic acid (DNSA) was used to carry out the α-amylase inhibition assay (Wickramaratne et al., 2016). The extract was dissolved in buffer (Na_2_HPO_4_/NaH_2_PO_4_) (0.02 M), NaCl (0.006 M) at pH 6.9 to provide concentrations extending from 1.9 to 1,000 μg/mL, and then in a minimum of 10% DMSO. Two hundred μL of extract were combined with 2 units/ml of α-amylase solution, and the mixture was then incubated at 30°C for 10 min. Each tube then received 200 μL of the starch solution, which contained 1% starch in water (w/v), and was left to sit for 3 min. Two hundred μL of the DNSA reagent (contained of 12 g of Rochelle salt that dissolved in 8 mL of 2 M NaOH and 20 mL of 96 mM 3 DNSA solution) were added to the reaction to stop it, and it was then boiled for 10 min at 85–90°C in a water bath. A UV-Visible Biosystem 310 spectrophotometer was utilized to measure the absorbance at 540 nm after the mixture had been cooled to room temperature and diluted with 5 ml of distilled water. By substituting 200 μL of buffer for the 200 μL of tested samples, an enzyme activity of 100% was used as a control. The tested samples were prepared at each concentration in a similar manner, but without the enzyme solution, to create a blank reaction. The following equation was used to calculate the percent inhibition of the α-amylase and to express the inhibitory activity as a percentage. The IC_50_ values were then calculated from the graph of the percent inhibition of the α-amylase against extract concentration.


$$A\,m\,y\,l\,a\,s\,e\,i\,n\,h\,i\,b\,i\,t\,i\,o\,n\%=\frac{100\%\,c\,o\,n\,t\,r\,o\,l\,A\,b-S\,a\,m\,p\,l\,e\,A\,b}{100\%\,c\,o\,n\,t\,r\,o\,l\,A\,b}\times 100$$

### 2.10. Statistical analysis

Three replicates of the experiments were performed to evaluate the standard deviation (SD). SD and variance were designed via SPSS ver. 22.0 software (version 14, IBM, Armonk, NY, USA). IC_50_ depending on GraphPad Prism^®^ software (version 5.0, Boston, USA), was calculated.

## 3. Result and discussion

### 3.1. Chemical characterization of dill extract and DELChNPs characterization

**FIGURE 2:**
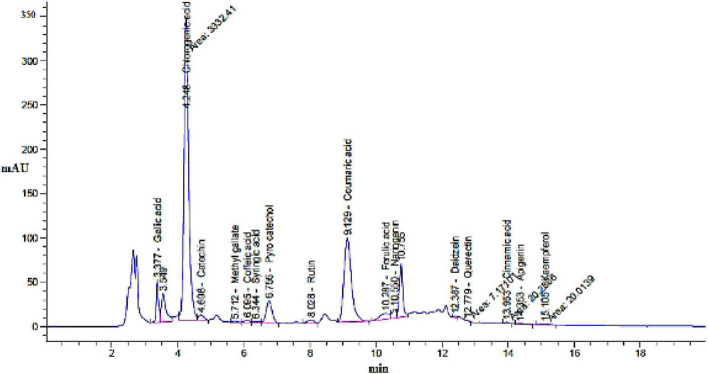
Chromatogram of flavonoid and phenolic constituents identified by HPLC in *Anethum graveolens* extract.

**FIGURE 3:**
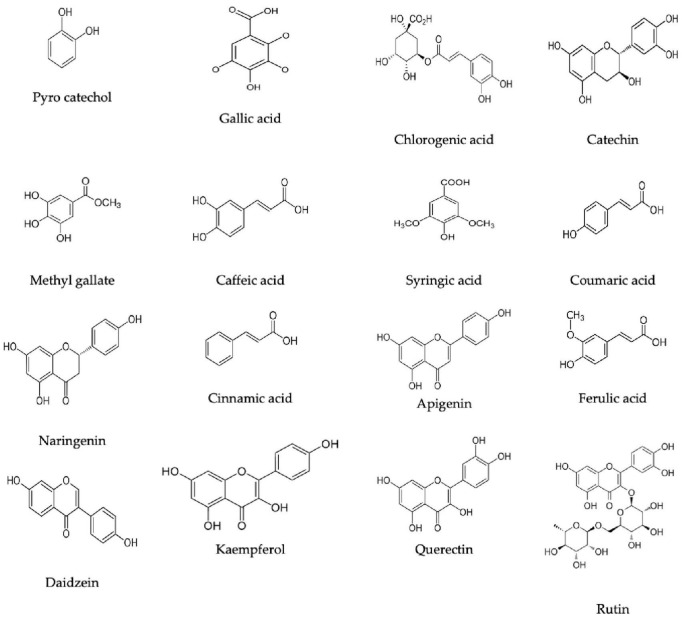
Flavonoid and phenolic constituents chemical formulas detected in *Anethum graveolens* extract.

**FIGURE 4:**
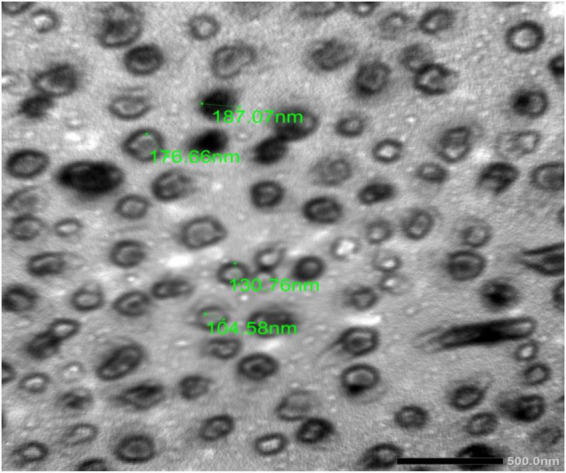
TEM DELChNPs.

Based on the HPLC analysis (Table 1; Figure 2), 18 compounds associated with phenolic and flavonoid were detected in dill extract (DE) with different area, area %, retention times, and molecular weight besides molecular formula (Figure 3). Chlorogenic acid was recognized in the highest concentration (22526.51 μg/mL) followed by coumaric acid (2236.21 μg/mL), and pyrocatechol (2113.81 μg/mL), while gallic acid and catechin were detected in moderate concentration 1013.10 μg/mL and 821.73 μg/mL. Other compounds were detected but in a lower concentration of less than 100 μg/mL, including cinnamic acid (18.45 μg/mL), methyl gallate (24.44 μg/mL), apigenin (32.62 μg/mL), daidzein (34.93 μg/mL), quercetin (46.19 μg/mL), syringic acid (58.06 μg/mL), and kaempferol (71.18 μg/mL). Naringenin and rutin, as important biological compounds, were also identified in the extract. In DE, two unknown compounds with different retention times and area (%) appeared in the chromatogram of HPLC, while it was found to be devoid of vanillin, ellagic acid, and hesperidin. Our results partially agreed with Ahmed et al. (2021), who recorded the absence of caffeic acid, ellagic acid, kaempferol, and apigenin but chlorogenic acid, catechin, rutin, and quercetin occurred in the extract of *A. graveolens*. These variances in the flavonoid and phenolic contents may arise from some genetic and ecological differences besides the nutritional and cultivation status of the plants, besides the extraction methods and the used solvents (Ghany, 2014). Most of the detected compounds, either phenolic or flavonoid, documented their validity in the various biological activities as mentioned in the scientific papers. Su et al. (2019) reported that chlorogenic acid inhibited the food-borne pathogen *Pseudomonas aeruginosa* causing leakage of intracellular materials through damage to the intracellular and outer membrane. Another study found that chlorogenic acid exerts an inhibitory action on the growth of *Salmonella enteritidis*, a food-borne species in chilled fresh chicken via disruption of cell metabolism (Sun et al., 2020). In addition, p-coumaric acid showed inhibitory activity against different species of bacteria (Lou et al., 2012). TEM indicated that the morphological and size of created DELChNPs indicated that the diameter of DELChNPs was between 100 and 200 nm (Figure 4).

**TABLE 1:** Flavonoid and phenolic constituents identified in *Anethum graveolens* extract.

Constituent	Retention time	Area	Area (%)	Concentration (μ g/mL)
Gallic acid	3.377	237.339	3.612	1013.10
Unknown	3.549	315.258	4.797	Undetected
Chlorogenic acid	4.248	3332.409	50.707	22526.51
Catechin	4.698	67.540	1.028	821.73
Methyl gallate	5.712	9.046	0.138	24.44
Caffeic acid	6.095	26.118	0.397	100.35
Syringic acid	6.344	16.820	0.256	58.06
Pyro catechol	6.756	303.180	4.613	2113.81
Rutin	8.028	49.676	0.756	288.67
Ellagic acid	8.903	0.000	0.000	0.00
Coumaric acid	9.129	1488.544	22.650	2236.21
Vanillin	9.808	0.000	0.000	0.00
Ferulic acid	10.287	131.859	2.006	441.60
Naringenin	10.550	90.847	1.382	529.69
Unknown	10.755	435.387	6.625	Undetected
Daidzein	12.387	11.526	0.175	34.93
Quercetin	12.779	7.171	0.109	46.19
Cinnamic acid	13.953	20.287	0.309	18.45
Apigenin	14.353	8.816	0.134	32.62
Kaempferol	15.105	20.014	0.305	71.18
Hesperetin	15.636	0.000	0.000	0.00

### 3.2. Antimicrobial activity of DE and DELChNPs

**FIGURE 5:**
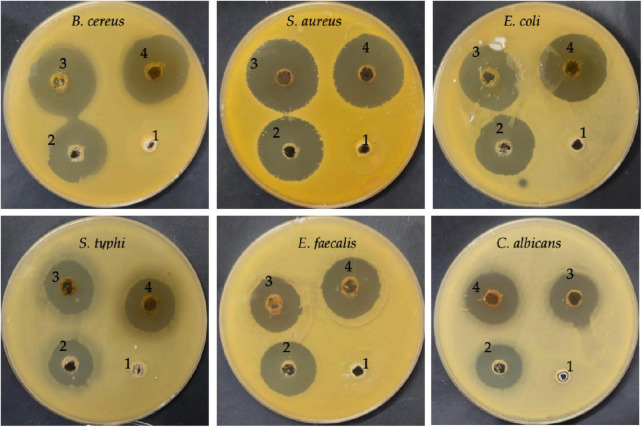
Antimicrobial activity of DE (3), DELChNPs (4), antibiotic/antifungal as a positive control (2), and chitosan (1) toward food-borne microorganisms via well diffusion assays.

The inhibitory activity of DE was examined alone and DE loaded with ChNPs (DELChNPs) using *B. cereus*, *S. aureus*, *E. coli*, *S. typhi*, *E. faecalis*, and *C. albicans*. Application of DELChNPs reflected high antimicrobial activity with inhibition zones 28.50 ± 0.87, 30.33 ± 0.58, 29.33 ± 0.58, 23.17 ± 0.76, 25.76 ± 0.58, and 24.17 ± 0.29 mm, compared to the observed inhibition zones 25.83 ± 1.44, 29.67 ± 0.85, 24.83 ± 0.76, 20.33 ± 1.53, 21.17 ± 0.29, and 19.67 ± 1.15 mm using DE against *B. cereus*, *S. aureus*, *E. coli*, *S. typhi*, *E. faecalis*, and *C. albicans*, respectively (Table 2; Figure 5). Ahmed et al. (2021) mentioned that DE had no influence on *Aspergillus fumigates* and *Staphylococcus aureus*, and *Proteus vulgaris* but proved the greatest influence on *Bacillus subtilis* and *Candida albicans*. Also, DE exhibited inhibitory activities against *Pseudomonas aeruginosa, Staphylococcus epidermidis*, and *Enterococcus faecalis* (Ozliman et al., 2021).

**TABLE 2:** Antimicrobial activity of *Anethum graveolens* extract, *Anethum graveolens* extract loaded with chitosan nanoparticles and chitosan toward food-borne microorganisms.

Food-borne microorganism	Inhibition zone (mm)			
	DELChNPs	DE	Chitosan only	Control
*B. cereus*	28.50 ± 0.87	25.83 ± 1.44	NA	24.33 ± 0.58
*S. aureus*	30.33 ± 0.58	29.67 ± 0.85	NA	28.17 ± 0.29
*E. coli*	29.33 ± 0.58	24.83 ± 0.76	NA	24.67 ± 1.15
*S. typhi*	23.17 ± 0.76	20.33 ± 1.53	NA	17.00 ± 0.50
*E. faecalis*	25.76 ± 0.58	21.17 ± 0.29	NA	25.33 ± 1.15
*C. albicans*	24.17 ± 0.29	19.67 ± 1.15	NA	18.17 ± 0.29

The encapsulation of the extract ChNPs provides a remarkable potential for boosting the activity of the extract alone against the growth of microorganisms. The antimicrobial activity of the DE alone and DELChNPs was compared with the activity of standard antibiotics against bacteria and antifungal against *C. albicans* which gave less inhibition zones in all tested microorganisms except *E. faecalis* (25.33 ± 1.15 mm), indicating that ChNPs enhanced the activity of DE. These findings are in agreement with the outcomes of Bagheri et al. (2021) which showed an improvement in the antibacterial activity of encapsulated nettle essential oil with ChNPs against food-borne bacteria. It has been described that NPs can interpenetrate through the bacterial cell membrane and injure their cell wall; so, they prove a better antimicrobial potential contrasted to larger particles. Karaaslan et al. (2021) indicated that encapsulation of natural compounds increased its efficacy against pathogenic microorganisms. In a recent study, Qanash et al. (2023a) mentioned the highest efficacy of ChNPs loaded with the extract of *Artemisia judaica* against human pathogenic microorganisms compared with the antimicrobial activity of *A. judaica* extract alone. *Helicobacter pylori* was severely inhibited using *Aloe vera* gel loaded with ChNPs in contrast to using the *Aloe vera* gel extract only (Yahya et al., 2022). These promising activities may be attributed to the unique properties of ChNPs, such as minor size and high surface charge, which enables the contact among the active extracts supported with ChNPs packed and cell wall of microorganisms.

In this context, the study continued with the detection of MIC and MBC of the DE alone and DELChNPs (Table 3). Through the implementation of that experiment, it is clear that the MIC of DELChNPs is very much less than the MIC of DE against all tested microorganisms. The values of MIC were 15.41 ± 0.36, 7.70 ± 0.17, 15.58 ± 0.07, 31.08 ± 0.07, 31.04 ± 0.07, and 62.33 ± 0.29 μg/mL using DELChNPs, while it was 62.33 ± 0.29, 31.08 ± 0.07, 62.50 ± 0.29, 31.08 ± 0.07, 31.04 ± 0.07, and 249.0 ± 1.73 μg/mL against *B. cereus*, *S. aureus*, *E. coli*, *S. typhi*, *E. faecalis*, and *C. albicans*, respectively. Also, by studying the MBC of the DE and DELChNPs, it was found that DELChNPs were more effective than DE against also all tested microorganisms, particularly *S. aureus* (7.73 ± 0.12 μg/mL and 62.33 ± 0.29 μg/mL, respectively), *S. typhi* (62.50 ± 1.0 μg/mL and 503.33 ± 5.77 μg/mL, respectively) and *E. faecalis* (31.23 ± 0.03 μg/mL and 246.67 ± 5.77 μg/mL, respectively). Our results matched with the outcomes of Qanash et al. (2023a), where the MIC value of *A. judaica* extract alone was higher (15.65, 62.5, 31.25, 15.62, and 31.25 μg/mL) than the MIC value (1.95, 0.97, 4.1, 3.9, and 15.62 μg/mL) of ChNPs loaded with the extract of *A. judaica* against *E. coli*, *B. subtilis*, *K. pneumonia*, *S. aureus*, and *C. albicans*, respectively. The efficacy of plant extract was classified according to Kuete (2010) as good, moderate, and weak if the MIC is less than 100 μg/mL, from 100 μg/mL to 625 μg/mL, and is more than 625 μg/mL, respectively. So, the DE, particularly DELChNPs, in the present study had good activity against the examined bacteria. The MBC/MIC index was calculated to detect the cidal or static potential of the DE and DELChNPs (Table 3). An index of more than 4 indicated its static potential, while less than 4 indicated its cidal effect. Therefore, MBC/MIC index described DELChNPs as having more promising effectiveness than DE.

**TABLE 3:** MIC and MBC of DE and DELChNPs against tested microorganisms.

Food-borne microorganism	MIC		MBC		MBC/MIC index DE	MBC/MIC index DELChNPs
	DE	DELChNPs	DE	DELChNPs		
*B. cereus*	62.33 ± 0.29	15.41 ± 0.36	122.33 ± 2.52	31.13 ± 0.07	1.96	2.02
*S. aureus*	31.08 ± 0.07	7.70 ± 0.17	62.33 ± 0.29	7.73 ± 0.12	2.01	1.00
*E. coli*	15.58 ± 0.07	62.50 ± 0.29	62.60 ± 0.17	248.33 ± 2.89	4.01	3.97
*S. typhi*	124.33 ± 1.15	31.08 ± 0.07	503.33 ± 5.77	62.50 ± 1.0	4.04	2.01
*E. faecalis*	125.33 ± 0.58	31.04 ± 0.07	246.67 ± 5.77	31.23 ± 0.03	1.97	1.01
*C. albicans*	249.0 ± 1.73	62.33 ± 0.29	500.0 ± 10.0	124.33 ± 1.15	2.01	1.99

**FIGURE 6:**
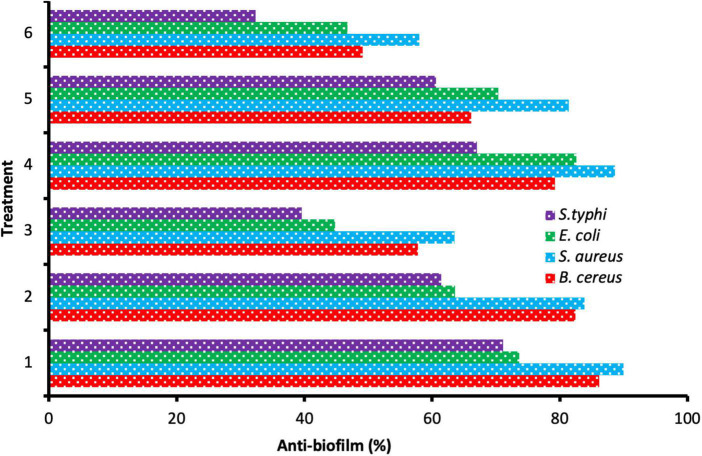
Anti-biofilm activity of different treatments 75% MBC DELChNPs (1), 50% MBC DELChNPs (2), 25% MBC DELChNPs (3), 75% of MBC DE (4), 50% of MBC DE (5), 25% of MBC DE (6) against different bacterial species.

Microbial biofilm remains a worldwide risk to health because of its great refractoriness to treatment and the capability to aggravate contamination and infections. Therefore, the search for new efficacious compounds to attack this problem is a priority (Nostro et al., 2016). Anti-biofilm activity was enhanced as a result of the application of ChNPs with the extract. Still, the level of the anti-biofilm activity differed based on the tested species of bacteria and the amount of MBC used (Figure 5). The result of the anti-biofilm activity at different quantities of MBC of the DE and DELChNPs was visualized in Figure 6. The addition of ChNPs to the DE had a clear effect on the bacterial potency via studying the anti-biofilm activity. For instance, the anti-biofilm activity against *B. cereus* was 49.12, 66.14, and 79.26% using 25, 50, and 75% of MBC DE, but the anti-biofilm activity became 57.74, 82.46, and 86.15% when the DE loaded by ChNPs, respectively. The same level of results in the case of *S. aureus* and *S. typhi* was similar, but *E. coli* was unlike *B. cereus*. Biofilm formation by *S. aureus* was the most sensitive to DE alone or DELChNPs at all levels of MBC, followed by *B. cereus* and *S. typhi*. These differences may be attributed to the various virulence factors in bacteria.

### 3.3. Antioxidant activity of DE and DELChNPs

The antioxidant activity of DE was reported alone and DELChNPs (Table 4). DPPH scavenging % was incremented with the increasing concentration in dependent mode at all treatments and extended to 92.4 and 93.6% using DE and DELChNPs, respectively. From the result, it’s clear that ChNPs promote the antioxidant activity of the DE, where at 1.95, 7.81, 31.25, 125, and 500 μg/mL, the DPPH scavenging % was 35.2, 50.5, 63.3, 76.4, and 88.3% using DELChNPs compared to 30.6, 44.1, 58.2, 71.5, and 85.1% using DE alone, respectively. Remarkably, 7.8 μg/mL was the value of IC_50_ of DELChNPs, while 13.96 μg/mL was the value of IC_50_ of DE alone compared with IC_50_ 4.83 μg/mL of ascorbic acid as a positive standard antioxidant drug. Our findings demonstrated the potential for the utilization of DE, particularly DELChNPs, as a safe antioxidant agent to improve cosmetic and therapeutic applicability. A previous study indicated that DE in the form of aqueous and ethanolic extract presented radical scavenging potential (Selen Isbilir and Sagiroglu, 2011) but with more IC_50_ values of 193.39 μg/mL and 475 μg/mL, respectively, than the obtained IC_50_ in the present study. In a recent study, inhibition of DPPH radicals was recorded using the ethanolic extract of aerial parts of DE with IC_50_ 173.04 μg/mL (Peerakam et al., 2022). The excellent potential of antioxidants associated with DE is recognized not only by the existence of phenolic and flavonoids but also by the presence of volatile constituents, as mentioned in other studies (Ramadan et al., 2013). Some literature indicated that the antioxidant activity of plant extracts was enhanced and acquired more stability when encapsulated with ChNPs (Kesharwani et al., 2019; Yahya et al., 2022).

**TABLE 4:** DPPH scavenging % of *Anethum graveolens* extract, *Anethum graveolens* extract loaded with chitosan nanoparticles and ascorbic acid.

Concentration (μ g/mL)	Extract			Extract +Chitosan			Ascorbic acid		
	OD mean	DPPH scavenging %	± SD	ODMean	DPPH scavenging %	± SD	OD mean	DPPH scavenging %	± SD
1,000	0.100	92.4	0.005	0.081	93.6	0.004	0.049	95.6	0.002
500	0.220	85.1	0.003	0.167	88.3	0.002	0.091	92.8	0.004
250	0.337	78.1	0.003	0.243	83.8	0.003	0.122	91.2	0.005
125	0.447	71.5	0.002	0.366	76.4	0.005	0.225	84.8	0.006
62.50	0.558	64.8	0.005	0.474	69.8	0.004	0.365	76.5	0.006
31.25	0.668	58.2	0.005	0.582	63.3	0.006	0.478	69.7	0.004
15.63	0.785	51.1	0.003	0.683	57.2	0.005	0.593	62.8	0.005
7.81	0.901	44.1	0.004	0.795	50.5	0.004	0.725	54.6	0.003
3.90	1.008	37.7	0.003	0.906	43.8	0.004	0.898	44.5	0.002
1.95	1.125	30.6	0.002	1.048	35.2	0.009	0.966	40.3	0.007
0	1.632	0.0	0.004	1.632	0.0	0.004	1.632	0.0	0.004
IC^50^	13.96 μg/mL			7.8 μg/mL			4.83 μg/mL		

### 3.4. Anti-diabetic activity of DE and DELChNPs

The obtained results indicated that DE possesses anti-diabetic activity *in vitro* via inhibition of amylase activity that raised with the rise of the extract concentration (Table 5). Unfortunately, there are slight differences between the effect of DE alone and DELChNPs on amylase inhibition %. The values of IC_50_ were 164.2 μg/mL and 164.3 μg/mL using DE alone and DELChNPs, respectively. These findings reflected the un-promoting effect of ChNPs on the activity of the extract. Goodarzi et al. (2016) reflected on the anti-diabetic activity of DE. HPLC analysis presented that DE had quercetin. According to Ebrahim Abbasi et al. (2012), these constituents have hypolipidemic and hypoglycemic properties. Yousaf and Shahid (2020) confirmed the hypolipidemic and hypoglycemic effects of the dried leaves of DE through its ability to minimize the level of glucose in the blood and treat the disorders of cardiovascular system.

**TABLE 5:** Effect of DE and DELChNPs on amylase inhibition.

Concentration (μ g/mL)	DE			DELChNPs		
	OD mean	Amylase inhibition %	± SD	OD mean	Amylase inhibition %	± SD
1,000	0.290	85.4	0.010	0.260	86.9	0.026
800	0.477	76.0	0.006	0.450	77.4	0.036
600	0.707	64.5	0.021	0.663	66.7	0.031
400	0.817	59.0	0.012	0.870	56.3	0.020
200	0.987	50.4	0.006	0.960	51.8	0.020
100	1.110	44.2	0.010	1.090	45.2	0.010
80	1.290	35.2	0.010	1.287	35.3	0.025
40	1.400	29.6	0.010	1.443	27.5	0.032
20	1.643	17.4	0.015	1.640	17.6	0.010
10	1.753	11.9	0.035	1.840	7.5	0.020
Control 100%	1.990	0.0	0.010	1.990	0.0	0.010
IC_50_	164.2 μg/mL			164.3 μg/mL		

## 4. Conclusion

Various constituents, including phenolic and flavonoid contents with various biological activities, were detected in DE via HPLC. The occurrence of different compounds such as chlorogenic acid, coumaric acid, pyrocatechol, gallic acid, catechin, cinnamic acid, methyl gallate, apigenin, daidzein, quercetin, syringic acid, kaempferol, naringenin, and rutin indicating its vital role in the biological system. Antimicrobial activity against food-borne microorganisms, including MIC, MBC, and anti-biofilm of DE were enhanced when DE was loaded with ChNPs. Also, antioxidant activity was enhanced when DE was loaded with ChNPs, but negligible enhancement in the anti-diabetic activity was observed through the detection of IC_50_. Therefore, the current investigation recommends the potential applications of DELChNPs via spraying on vegetables or food products to eliminate foodborne pathogens.

## Data availability statement

The original contributions presented in this study are included in the article/supplementary material, further inquiries can be directed to the corresponding author.

## Author contributions

AM contributed to the conception and design of the study as well as organized the database, performed the statistical analysis, wrote the first draft of the manuscript, revised it, and read and approved the submitted version.
